# A Comparative Study of Food Source Selection in Stingless Bees and Honeybees: Scent Marks, Location, or Color

**DOI:** 10.3389/fpls.2020.00516

**Published:** 2020-05-06

**Authors:** Sebastian Koethe, Vivian Fischbach, Sarah Banysch, Lara Reinartz, Michael Hrncir, Klaus Lunau

**Affiliations:** ^1^Institute of Sensory Ecology, Heinrich Heine University Düsseldorf, Düsseldorf, Germany; ^2^Departamento de Biociências, Universidade Federal Rural do Semi-Árido, Mossoró, Brazil; ^3^Instituto de Biociências, Universidade de São Paulo, São Paulo, Brazil

**Keywords:** eusocial bees, chemical cues, color cues, location-dependent cues, foraging behavior

## Abstract

In social bees, the choice of food sources is based on several factors, including scent marks, color, and location of flowers. Here, we used similar setups, in which two stingless bee species, *Melipona subnitida* and *Plebeia flavocincta*, and the Western honeybee, *Apis mellifera*, were tested regarding the importance of chemical cues, color cues, and location-dependent cues for foraging behavior. It was determined whether workers chose food sources according to (1) scent marks deposited by conspecifics, (2) the color hue of a food source, (3) the trained location or the proximity of a food source to the hive. All three species preferred the scent-marked over an unmarked feeder that was presented simultaneously, but *M. subnitida* showed a weaker preference compared to the other species. When trained to blue feeders all three bee species preferred blue, but *A. mellifera* showed the strongest fidelity. The training to yellow feeders led to less distinct color choices. Only workers of *M. subnitida* mostly orientated at the training position and the close proximity to the nest. Whether the distance of a feeding site influenced the choice was dependent on the tested parameter (color or scent marks) and the species. Workers of *M. subnitida* preferably visited the feeder closer to the nest during the scent mark trials, but choose randomly when tested for color learning. Worker honeybees preferred the closer feeding site if trained to yellow, but not if trained to blue, and preferred the more distant feeder during the scent mark trials. Workers of *P. flavocincta* preferred the closer feeder if trained to blue or yellow, and preferred the more distant feeder during the scent mark trials. The disparity among the species corresponds to differences in body size. Smaller bees are known for reduced visual capabilities and might rely less on visual parameters of the target such as color hue, saturation, or brightness but use scent cues instead. Moreover, the dim-light conditions in forest habitats might reduce the reliability of visual orientation as compared to olfactory orientation. Honeybees showed the most pronounced orientation at floral color cues.

## Introduction

Foraging bees use visual and olfactory cues to find and select food sources and deploy innate or learned preferences to detect flowers (Lunau and Maier, 1995; Dyer et al., 2016). Primarily, a forager’s choice is biased by innate preferences for particular colors, shapes, and odors (Menzel, 1967; Giurfa et al., 1995; Lehrer et al., 1995; Lunau et al., 1996; Gumbert, 2000; Biesmeijer and Slaa, 2004; Raine and Chittka, 2007; Howard et al., 2019). These innate preferences differ among species. In several experiments, preferences for specific hues and saturation of colors could be found for honeybees and bumble bees (Lunau, 1990; Giurfa et al., 1995; Lunau et al., 1996; Papiorek et al., 2013; Rohde et al., 2013), while stingless bees sparsely show preferences for color hue or saturation (Spaethe et al., 2014; Dyer et al., 2016; Koethe et al., 2016, 2018).

With increasing foraging experience, initial individual preferences may be either consolidated or modified through associative learning (Gumbert, 2000; Sánchez et al., 2008; Roselino et al., 2016). For instance, species-specific chemical footprints deposited by bees while landing on and manipulating flowers indicate the recent presence of a forager to subsequent visitors (Hrncir et al., 2004; Jarau et al., 2004; Eltz, 2006; Saleh and Chittka, 2006; Witjes et al., 2011). An initial attraction toward the familiar scent of conspecifics (Schmidt et al., 2005) may be reinforced when individuals learn to associate the footprints with high reward levels or reversed when scent marks indicate depleted flowers (Saleh and Chittka, 2006; Roselino et al., 2016).

Learning and memory play a major role in bee foraging, enabling the repeated visit to sustainable food sources (Breed et al., 2002; Reinhard et al., 2004, 2006; Jesus et al., 2014), flower constancy (Free, 1963; Biesmeijer and Toth, 1998; Slaa et al., 1998, 2003), and the discovery of new patches of known food plants (Biesmeijer and Slaa, 2004). In addition to memorizing scent and location of resources (Reinhard et al., 2004, 2006), bees learn both color and position of landmarks, which facilitates the orientation toward food sources and the nest (Cartwright and Collett, 1983; Cheng et al., 1986, 1987; Chittka et al., 1995; Menzel et al., 2005). However, species differ concerning their learning ability (Pessotti and Lé’Sénéchal, 1981; Mc Cabe et al., 2007), which might be associated with differences in life-history and ecological traits among bee species, such as longevity of individuals (Ackerman and Montalvo, 1985), the degree of floral specialization (Cane and Snipes, 2006), and food niche-breath (Biesmeijer and Slaa, 2006).

In eusocial bees, including the stingless bees (Meliponini), bumble bees (Bombini), and honeybees (Apini), food source selection is not only based on individual foraging preferences, but relies to a large extent on social information. On their return to the nest, foragers transmit olfactory and gustatory information about the exploited food source to nestmates, which biases the subsequent food choice of the receivers (Farina et al., 2005, 2007; Mc Cabe and Farina, 2009). Moreover, returning foragers of many species announce the existence of lucrative food sources through thoracic vibrations (stingless bees: Lindauer and Kerr, 1958; Esch et al., 1965; Barth et al., 2008; Hrncir and Barth, 2014; honeybees: Esch, 1961; Waddington and Kirchner, 1992; Hrncir et al., 2011). Inactive individuals may use these mechanical signals for their decision of whether to engage in foraging or to remain in the nest. In addition, foragers of some eusocial bee species guide the recruits to the location of the exploited food patch. Honeybees (all species) use an elaborated dance language (waggle dance) communicating information about distance, direction, and quality of foraging sites (Von Frisch, 1967; Dyer, 2002). Stingless bees (few species), in contrast, lay polarized trails of species-specific pheromone marks that guide recruits with high precision toward the goal (Lindauer and Kerr, 1958; Schmidt et al., 2003; Nieh et al., 2004; Barth et al., 2008; Jarau, 2009). At the food patch, foraging choices are influenced by field-based social information, like olfactory footprints and the visual presence of con- or heterospecific foragers (Slaa et al., 2003). Depending on the composition of the foraging community at the food patch, these passively provided cues may cause local enhancement or local inhibition (Slaa and Hughes, 2009). Thus, food source selection in eusocial species is based on a complex interplay between individual preferences and social information.

Differences among social bee species regarding ecological (habitat, food niche), physiological (learning ability, visual capacity, color vision), and behavioral features (innate preferences, foraging strategy, recruitment mechanism) may result in differences concerning the parameters used in foraging decisions. With more than 500 described species, stingless bees (Meliponini) are the most speciose group of eusocial bees with very diverse characteristics regarding body size, colony size, nesting biology, brood cell arrangement, queen production, foraging strategies, and recruitment mechanisms (Michener, 1974, 2013; Johnson, 1983; Wille, 1983; Engels and Imperatriz-Fonseca, 1990; Roubik, 2006; Barth et al., 2008). Given this biological diversity, we can expect differences concerning the mechanisms of food source selection among species. In the present study, we investigated the food source selection by two stingless bee species, *Melipona subnitida* and *Plebeia flavocincta*, and the Western honeybee, *Apis mellifera*. Since stingless bees show only weak preferences for colors compared to other bee species (Dyer et al., 2016; Koethe et al., 2016, 2018), alternative parameters could be of importance for foraging choices. Of interest were the roles of scent marks (olfactory footprints), the color, and the location of a food source. *Melipona* species are known to mark food sources with olfactory footprints (Jarau, 2009; Roselino et al., 2016). For *P. flavocincta*, no specific information concerning scent communication is available so far (Aguilar et al., 2005). However, given that all bee species studied to this moment deposit chemical footprints at food sources (Goulson et al., 1998; Eltz, 2006; Yokoi et al., 2007; Jarau, 2009; Witjes et al., 2011), scent cues can also be postulated for this meliponine species. *A. mellifera* is known for marking food sources directly (Giurfa and Nunez, 1992).

The aim was to analyze how the three investigated social bee species use the parameters color, scent marks, or location differently during the colony foraging processes. We test the hypothesis that these bees possess a hierarchy in the use of the parameters color, scent marks, and location of flowers. We expect honeybees to rely more on color cues than the two stingless bee species. For the two stingless bee species, we assume that they follow scent markings of conspecifics more reliable than honeybees. Since small stingless bee might exploit nectarrich flowers by repeated visits to the same individual flower, we assume that the location of the flower is of higher importance in the smaller bees.

## Materials and Methods

This study is part of a research project on color preferences in stingless bees conducted in Australia and Brazil (Köthe, 2019).

### Study Site and Bee Species

The foraging behavior of the stingless bee species was investigated at the Brazilian Federal University at Mossoró (Universidade Federal Rural do Semi-Árido), located in the Brazilian tropical dry forest, the Caatinga at 5°12′13.3″S 37°19′44.8″W. For our experiments, we used two stingless bee species native to the study region, *M. subnitida* (six colonies) and *P. flavocincta* (one colony) (Zanella, 2000; Imperatriz-Fonseca et al., 2017). *P. flavocincta* is the smallest bee with less than 5 mm body length (Cockerell, 1912), *M. subnitida* is intermediate with 7.5–8.5 mm (Schwarz, 1932), and *A. mellifera* is the largest with more than 11 mm (Amiet and Krebs, 2012). Colonies of the stingless bee species were kept in wooden nest-boxes at the university’s meliponary (Meliponário Imperatriz) and were freely foraging. The foraging behavior of the Western honeybee, *A. mellifera*, was studied at the botanical garden of the Heinrich Heine University Düsseldorf, Germany at 51°11′10.7″N 6°48′14.1″E. Foragers of five nests were trained to participate in the experiment. The colony size of the three tested species differs and ranges from several thousand individuals (20.000–80.000) in a single colony of *A. mellifera* to several hundred (up to 1000) in *M. subnitida* (Wilson, 1971; Michener, 1974). For *P. flavocincta*, colony size has not been determined yet, but in other *Plebeia* species, colony size has been shown to range from 2.000 to 3.000 individuals (Roldão-Sbordoni et al., 2018).

The reasons for conducting the study on stingless bees and honeybees at different study sites were as follows: Most experimental research in Western honeybees has been done in Europe and Australia, excluding the Africanized honeybees available in Mossoro. Moreover, *A. mellifera* is not native in South America. Thus, direct comparison with literature data is easier when working with European Western honeybees, although direct comparison of foraging strategies in stingless bees and honeybees in the same habitat might also yield interesting results (Roubik and Buchmann, 1984). The origin of the Western honeybee is in the Middle East or Africa (Han et al., 2012) and Western honeybees have developed adaptations to get along with temperate climates (Han et al., 2012).

### Bee Training

For all tests and bee species, the training was identical. Workers of all three species were trained to mass feeders offering sugar solution (50%) affixed to tripods. The training to the mass feeders started at the respective nest’s entrance. After more than 10 workers regularly foraged at the feeder, it was moved in short steps (∼1 m) away from the nest until a distance of 15 m (site 1) or 17 m (site 2) was reached. Once at the final feeding site, the mass feeder was replaced by a colored gravity feeder (10 cm diameter, 5 cm height) that was used during the experiment. The gravity feeders were either blue (edding permanent spray RAL5010 enzianblau, edding International GmbH, Ahrensburg, Germany) or yellow (only for the color test; edding permanent spray RAL 1037 sonnengelb, edding International GmbH, Ahrensburg, Germany). The colors were measured using spectrometer analysis (USB4000 miniature fiber optic spectrometer, Ocean Optics GmbH, Ostfildern, Germany) at an angle of 45° using a UV-NIR deuterium halogen lamp (DH-2000-BAL, Ocean Optics GmbH), which was connected to the spectrometer by a UV–VIS fiber optic cable (Ø 600 μm, QR600-7-UV 125 BX, Ocean Optics GmbH). To calibrate the spectrometer, a black standard (black PTFE powder, Spectralon diffuse reflectance standard SRS-02-010, reflectance factor of 2.00%, Labsphere, Inc., North Sutton, NH, United States) and a white standard (white PTFE powder, Spectralon diffuse reflectance standard SRS99-010, reflectance factor of 99.00%, Labsphere, Inc., North Sutton, NH, United States) were used (Supplementary Figure S1). After the workers accepted the colored gravity feeder (henceforth “feeder”), a training period of 30 min started, in which the bees were allowed to forage *ad libitum* (approximate number of foragers during training phase*: M. subnitida* ≈ 10 individuals; *P. flavocincta* ≈ 30–50 individuals, *A. mellifera* ≈ 30–50 individuals). Workers were not marked during the training to keep the disturbance at the feeder to a minimum. Hence, no discrimination between experienced and inexperienced workers was possible.

### Experiments

#### Testing the Impact of Scent Marks

We conducted experiments investigating the influence of scent marks deposited at the training feeder on the choice behavior of foragers. For this experimental series, we used only blue-colored feeders. In total, we performed three trials with each bee species. In preliminary studies, this approach turned out to be most reasonable for comparative studies between these bee species. Each trial consisted of three sets of a 30-min training phase and a subsequent 5-min test phase, switching the feeder positions in pseudo-randomized order (SM1–SM3; Supplementary Table S1). After the training phase, we offered the incoming bees both the training feeder (scent-marked) and a clean blue-colored feeder (unmarked), one at each feeding site (Supplementary Table S1). During this test phase, both feeders contained sugar solution (50%). In total, we performed three trials of this experimental series with each bee species. A trial consisted of three pairs of a 30-min training phase and a 5-min test phase intermitted by 30-min training phases (SM1–SM32; Supplementary Table S1), switching the feeder positions in pseudo-randomized order. The three different bee species (*A. mellifera*, *M. subnitida*, and *P. flavocincta*) were tested separately. Workers that visited the feeder were either marked with nail polish on their first visit (*A. mellifera* and *M. subnitida*) or caught after landing (*P. flavocincta*) and released at the end of the respective 5-min test phase. Workers were allowed to participate in all three trials. To avoid pseudo-replication (*A. mellifera*, *M. subnitida*), only the first landing of an individual in each test phase was considered for the analysis. During the third test, all foragers were captured and killed by freezing to avoid pseudo-replication.

#### Testing the Impact of Color

In the second experimental series, we investigated the impact of color on the choice of food sites by workers. In this experimental series, we performed two different trial series with each bee species. Each trial consisted of two sets of a 30-min training phase and a subsequent 5-min test phase, switching the feeder positions in pseudo-randomized order (Supplementary Table S1). After the training phase (training feeder either blue or yellow; Supplementary Table S1), the training feeder was removed, and we offered the incoming bees a blue- and a yellow-colored feeder during the test phase, one at each feeding site (Supplementary Table S1). In trial series 1 (C1–C4; Supplementary Table S1), bees were trained to blue feeders in the first three training phases and a yellow feeder in the fourth (training to blue, retraining to yellow). In trial series 2 (C5–C8; Supplementary Table S1), foragers were trained to yellow feeders during three training phases and a blue feeder in the last training phase (training to yellow, retraining to blue). For the test phases, we used alcohol-cleaned feeders to eliminate the influence by any potential scent marks. During the test phase, both feeders offered sugar solution (50% weight on weight). Each trial series was repeated three to five times with different individuals. The bee species (*A. mellifera*, *M. subnitida*, and *P. flavocincta*) were tested separately and workers that visited the feeder were either marked with nail polish (*A. mellifera* and *M. subnitida*) or caught after landing on a feeder (*P. flavocincta*) and released at the end of the respective 5-min test phase. To avoid pseudo-replication (*A. mellifera*, *M. subnitida*), only the first landing of an individual in each test was considered for the analysis. During the fourth test, all workers were captured and killed by freezing.

#### Testing the Impact of Location

To test whether bees visited the feeding site closer to the nest (site 1, 15 m) more often than the farther feeding site (site 2, 17 m) the results of all above described tests (scent marks and color) were analyzed concerning the influence of distance.

### Statistics

The statistical program R was used to analyze the data (R Development Core Team, 2019). The data were analyzed by testing the bees’ choices (the first decision of each test) for the different parameters (scent marks, color, distance) using a generalized linear mixed model (GLMM). We used the “lme4” package of R to analyze choices of the bees, which were assessed using GLMM with Poisson distribution of data (Bates et al., 2009; R Development Core Team, 2019). We analyzed the number of choices for each test as fixed effect and the position of the stimuli were used as random effect of the model when testing the influence of color and scent marks, while these parameters were used as random effect when testing the impact of distance on the bees’ choice behavior.

## Results

**FIGURE 1:**
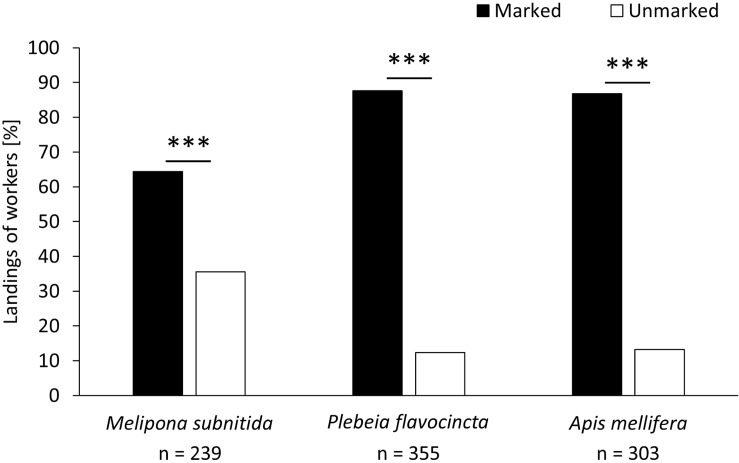
Landings of workers on a scent-marked and an unmarked feeder. A generalized linear mixed model was used for statistical analysis (^∗∗∗^*p* < 0.001).

In the first experimental series (influence of scent marks), foragers of all three bee species significantly preferred the previously visited training feeder over the clean feeder (Figure 1; *M. subnitida*: *n* = 239, *z*-value = −8.346, *p* < 0.001; *P. flavocincta*: *n* = 355, *z*-value = −12.15, *p* < 0.001; *A. mellifera*: *n* = 303, *z*-value = −10.46, *p* < 0.001).

**FIGURE 2:**
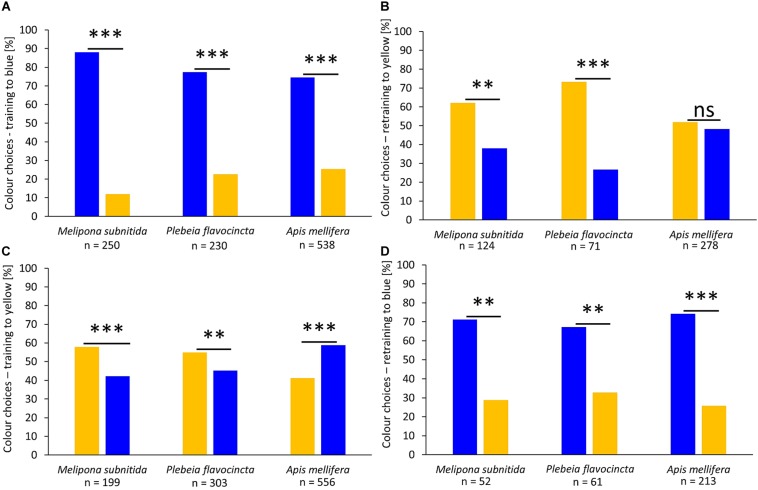
Color choices after training sessions. The three tested bee species were trained to forage on either a blue feeder **(A)** or a yellow feeder **(C)**. Furthermore, the workers were retrained to the opposite color **(B,D)**. A generalized linear mixed model was used for statistical analysis (^∗∗^*p* < 0.01; ^∗∗∗^*p* < 0.001; ns = not significant *p* > 0.05).

In the second experimental series, we investigated the influence of color on the feeder choice by the three bee species. After training to a blue-colored feeder, all three species significantly preferred the blue feeder over the yellow feeder (Figure 2A; *M. subnitida*: *n* = 250, *z*-value = −10.24, *p* < 0.001; *P. flavocincta*: *n* = 230, *z*-value = −8.821, *p* < 0.001; *A. mellifera*: *n* = 538, *z*-value = −10.85, *p* < 0.001). When these workers were retrained to forage on a yellow feeder during the last training phase, the two stingless bee species significantly preferred the yellow feeder while honeybee workers visited both colors equally (Figure 2B; *M. subnitida*: *n* = 124, *z*-value = 2.667, *p* = 0.007; *P. flavocincta*: *n* = 71, *z*-value = 3.756, *p* < 0.001; *A. mellifera*: *n* = 278, *z*-value = 0.6, *p* = 0.549). When workers were initially trained to a yellow-colored feeder, both stingless bee species preferred the yellow feeder significantly over the blue feeder during the test, while *A. mellifera* preferred the blue feeder (Figure 2C; *M. subnitida*: *n* = 199, *z*-value = 3.318 *p* < 0.001; *P. flavocincta*: *n* = 303, *z*-value = 3.141, *p* = 0.002; *A. mellifera*: *n* = 556, *z*-value = 5.863, *p* < 0.001). Retraining to a blue feeder in the last training phase lead to a significant preference of the blue colored feeder in all three species (Figure 2D; *M. subnitida*: *n* = 52, *z*-value = −2.95, *p* = 0.003; *P. flavocincta*: *n* = 61, *z*-value = −2.632, *p* = 0.008; *A. mellifera*: *n* = 213, *z*-value = −6.74, *p* < 0.001).

**FIGURE 3:**
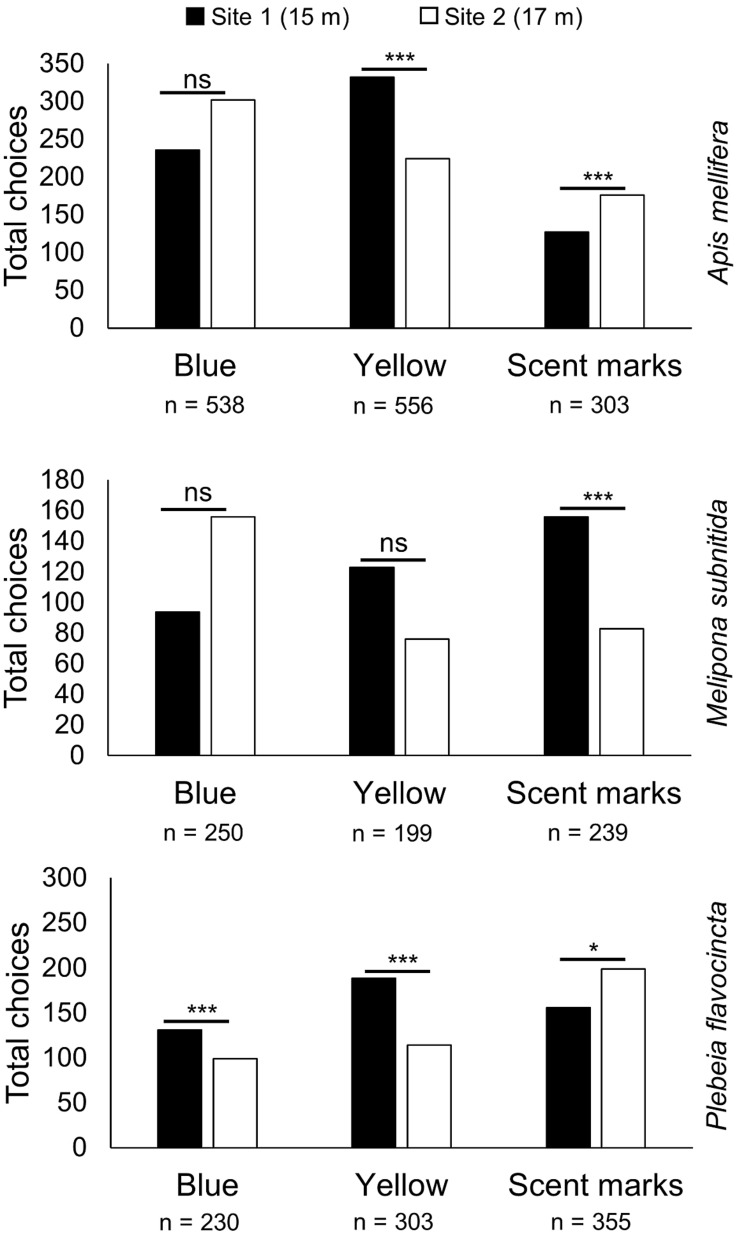
Landings of workers depending on the feeding site. The number of landings at the feeding sites with 15 m (site 1, black) and 17 m (site 2, white) distance to the hive were compared for *A. mellifera*, *M. subnitida*, and *P. flavocincta* for the two color trials, blue and yellow, and the scent mark trial (generalized linear mixed model *p < 0.05; ***p < 0.001; ns = not significant *p* > 0.05).

When analyzing the influence of the feeders’ positions on the food source choice, we observed that *M. subnitida* visited the feeding site closer to the nest during the scent mark trials (site 1, 15 m) significantly more often than the farther site (site 2, 17 m) (Figure 3; *n* = 239, *z*-value = −8.467, *p* < 0.001), while choosing randomly when tested for color differences (Figure 3; blue: *n* = 250, *z*-value = −0.045, *p* = 0.964; yellow: *n* = 199, *z*-value = 1.502, *p* = 0.133). Workers of *A. mellifera* significantly preferred the closer feeding site when they were trained to yellow (Figure 3; *n* = 556, *z*-value = −6.147, *p* < 0.001), but did not distinguish between the two sites when trained for blue (Figure 3; blue: *n* = 538, *z*-value = −0.951, *p* = 0.342; scent marks: *n* = 199, *z*-value = −1.502, *p* = 0.133). In the trial concerning scent marks workers of *A. mellifera* significantly preferred the farther away feeding site (Figure 3; *n* = 303, *z*-value = −10.46, *p* < 0.001). Workers of *P. flavocincta* preferred the closer feeding site when trained to blue or yellow (Figure 3; blue: *n* = 230, *z*-value = −5.036, *p* < 0.001; yellow: *n* = 71, *z*-value = −4.856, *p* < 0.001) and significantly visited the farther site when tested concerning scent marks (Figure 3; *n* = 355, *z*-value = −2.548, *p* = 0.011).

## Discussion

The results of this study show that the response to the color of feeder, scent marks, and locations differs among the tested species *P. flavocincta*, *M. subnitida*, and *A. mellifera*. Our results confirm previous findings about the important role of color for food plant detection in honeybees and add further findings to the diverse and sometimes less important role of color for food plant detection in stingless bees. In previous studies of color preferences in stingless bees, the results varied among species. While three species of the genus *Melipona* chose colors poorly, *Tetragonula carbonaria* chose colors according to their hue and *Partamona helleri* showed similar color choices as *A. mellifera* preferring spectrally purer colors and bluish color hues (Rohde et al., 2013; Dyer et al., 2016; Koethe et al., 2016, 2018). Particularly for the honeybee, it has been shown that besides innate preferences, absolute or differential conditioning and behavioral plasticity play important roles in how they exploit color information (Reser et al., 2012), and that strong color preferences impede learning of other features (Morawetz et al., 2013).

Workers of *A. mellifera* orientated most strongly according to colors. The blue-colored feeder was preferred in all tests with exception of the retraining to yellow, where *A. mellifera* showed no depicted choice for one of the two colors and the two stingless bee species preferred the yellow feeder. This is in accordance to previous studies showing that *A. mellifera* prefers blue colors over other color hues (Giurfa et al., 1995; Horridge, 2007). The two stingless bee species chose feeders according to their colors but rather preferred the feeder color of the previous training. Only when initially trained to yellow they showed weak (*M. subnitida*) or no preferences for the trained color (*P. flavocincta*). This preference for blue is in accordance with previous results of stingless bees, but also suggests that it is weaker in stingless bees than in honeybees (Dyer et al., 2016; Koethe et al., 2016). An explanation for less visually driven behavior in stingless bees could be the size differences compared to honeybees. *P. flavocincta* reaches a body size of 3.6–4.1 mm, *M. subnitida* of 7.5 mm, and *A. mellifera* is the largest of the three species with 13–16 mm (Hrncir and Maia-Silva, 2013; Maia-Silva et al., 2015; Imperatriz-Fonseca et al., 2017). Especially the size of the eyes, which is associated with body size, can impact the visual capacities of bees (Streinzer et al., 2016). Workers of *P. flavocincta* are rather small; consequently, their eyes are also small leading to poorer visual capabilities.

Both stingless bees and honeybees use scent cues to evaluate reward availability of food resources (Nunez, 1967; Butler et al., 1969; Ferguson and Free, 1979; Free and Williams, 1983; Corbet et al., 1984; Giurfa and Nunez, 1992; Giurfa, 1993; Stout et al., 1998; Williams, 1998; Stout and Goulson, 2001). In this study, all three species showed preferences for the marked feeder over the unmarked one. *P. flavocincta* and *A. mellifera* chose the marked feeder consistently (∼88% of choices), while *M. subnitida* preferred the marked feeder, but visited it less frequently (∼64% of choices).

*Plebeia flavocincta* was the only species that significantly preferred the closer feeding site when tested concerning colors and the farther feeding site when tested regarding scent marks. One interpretation is that *P. flavocincta* does not differentiate between colors and choses the closer feeding site, while the preference during the scent mark trial could be based on the fact that the scent marked feeder was positioned twice at the farther site and only once at the closer site. In contrast, *M. subnitida* was the only species in the scent mark trials that visited the food site with shorter distance to the hive more frequently. It seems likely that *M. subnitida* orientates on location rather than on scent marks. Previous studies showed that species of the genus *Melipona* mark food sites directly and do not lay scent trails (Hrncir et al., 2004). In order to recruit new foragers, it seems possible that *M. subnitida* relies strongly on piloting—leading new foragers from hive to food site during flight (Nieh et al., 2003). Foragers of *M. subnitida* could be observed to frequently arrive in small groups, while *A. mellifera* and *P. flavocincta* workers seemed more independent from each other. Scent marks play an important role for the communication of reward availability, but their impact on recruitment seems dependent on the specific strategy used by species (Free and Williams, 1983; Corbet et al., 1984; Giurfa and Nunez, 1992; Giurfa, 1993; Stout et al., 1998; Stout and Goulson, 2001; Schmidt et al., 2003). The attractiveness of scent marks, whether or not they were used for recruitment purposes, appears to be strong because scent-marked feeders were preferred by all three tested bee species. During the experiments workers foraged in groups and could be influenced by the presence of other individuals. An influence by social facilitation (Wilson, 1971) could not be excluded during the experiments, but when comparing the results for choices of blue and yellow feeders, after the respective training, an influence solely by the presence of conspecifics seems unlikely.

Another aspect that can explain the diverse results for the three tested bee species could be their natural habitat. *M. subnitida* originates from the Caatinga, which is an open habitat, while *P. flavocincta* inhabits a spacious habitat that extends from the Caatinga to the Atlantic Rainforest, which is a densely vegetated forest (Imperatriz-Fonseca et al., 2017). Because of its domestication, the honeybee is widespread all over the world. It originates from diverse habitats of Europe, the Middle East, and Africa. Open habitats are brightly illuminated, while forest habitats are characterized by dim-light conditions (Endler, 1992). Based on the light conditions of their respective habitat, it appears to be possible that *M. subnitida* and *A. mellifera* could rely to a greater extent on visual signals than *P. flavocincta* that encounters dim-light conditions and a less visually structured vegetation. In a densely vegetated habitat, scent marks could be a more reliable signal to guide foragers to a food source. Furthermore, temperate and sub-tropical regions experience more distinct seasons concerning weather conditions and the rhythm of flowering plants is directly influenced, while tropical and semi-arid regions have more steady weather conditions but are challenging for their inhabitants because of high temperatures (Prado, 2003; Zanella and Martins, 2003; Machado and Lopes, 2004; Maia-Silva et al., 2012, 2015; Hrncir et al., 2019).

Social bee species that face seasonal variations mass-collect floral resources for provision of the hive (Ramalho, 2004). These variations in floral resource availability could be another explanation for more distinct preferences for visual signals in honeybees when compared to tropical species, like *M. subnitida* and *P. flavocincta*, because only honeybees face strong seasonal variations (Michener, 1974; Kleinert-Giovannini, 1982; Roubik, 1982a; Seeley, 1985). Nonetheless, this would not explain the differences between *M. subnitida* and *P. flavocincta*.

## Conclusion

The three tested bee species reacted vaguely similar to color, scent marks, and location of food sources, but their main focus varies: While *A. mellifera* choose food sites according to both color and scent marks, *M. subnitida* orientates on location and color of food sites, and *P. flavocincta* relies mainly on scent marks. These variations are possibly based on different recruitment mechanisms (e.g., waggle dance of honeybees vs. piloting, excited movements, vibration, and scent mark deposition by stingless bees) or they could be the result of adaptations to the bees’ respective habitat and obliged morphological constraints. Although highly eusocial stingless and honeybees do not communicate the color of flowers to nestmates (Michelsen, 2014), flower color has a large impact on foraging decisions. This impact is demonstrated by the results of this study, that bees exhibit a spontaneous response to color cues and that they memorize color cues following experience; spontaneous response of bees and discrimination after conditioning might rely on different color parameters, such as color saturation and color hue (Rohde et al., 2013). Flower color has also been identified as a floral filter excluding bees from visiting the less preferred flower colors, i.e., red, UV-absorbing and white, UV-reflecting hummingbird-pollinated flowers (Lunau et al., 2011). Stingless bees are known as nectar robbers of hummingbird-pollinated flowers (Roubik, 1982b); it remains to be tested if the less pronounced color preferences in stingless bees are helpful for finding food on flowers displaying colors that are not adapted to bee-color vision and color preferences.

## Data Availability Statement

All datasets generated for this study are included in the article/Supplementary Material.

## Author Contributions

KL conceived and developed the research concept and supervised the study. SK designed the experiments. MH and KL supported the design of the experiments. SK, VF, LR, and SB conducted the experiments and collected and analyzed the data. SK wrote the manuscript. SB, VF, LR, SB, MH, and KL supported the writing of the manuscript.

## Conflict of Interest

The authors declare that the research was conducted in the absence of any commercial or financial relationships that could be construed as a potential conflict of interest.

## Supplementary Material

FIGURE S1 Spectral reflectance curves of colored feeders.

TABLE S1 Position of feeders in training and test phase. The order of tests was pseudo-randomized to ensure no influence of test order on the decisions of workers. Two experimental trials were conducted comprising four choice experiments each. C1–C4 are the tests which focused on blue color, while the tests C5–C8 focused on yellow color. SM1–3 = are tests which analyzed the impact of scent marks; m = marked feeder; u = unmarked feeder; C1–8 = are the tests analyzing the impact of color; b = blue; y = yellow; site 1 = 15 m distance to the hive; site 2 = 17 m distance to the hive.
