# Supplementary figures and images for: A Comparative Study of Food Source Selection in Stingless Bees and Honeybees: Scent Marks, Location, or Color
Source: Front Plant Sci. 2020 May 6;11:516. doi: 10.3389/fpls.2020.00516 (PMC7218124; doi:10.3389/fpls.2020.00516)

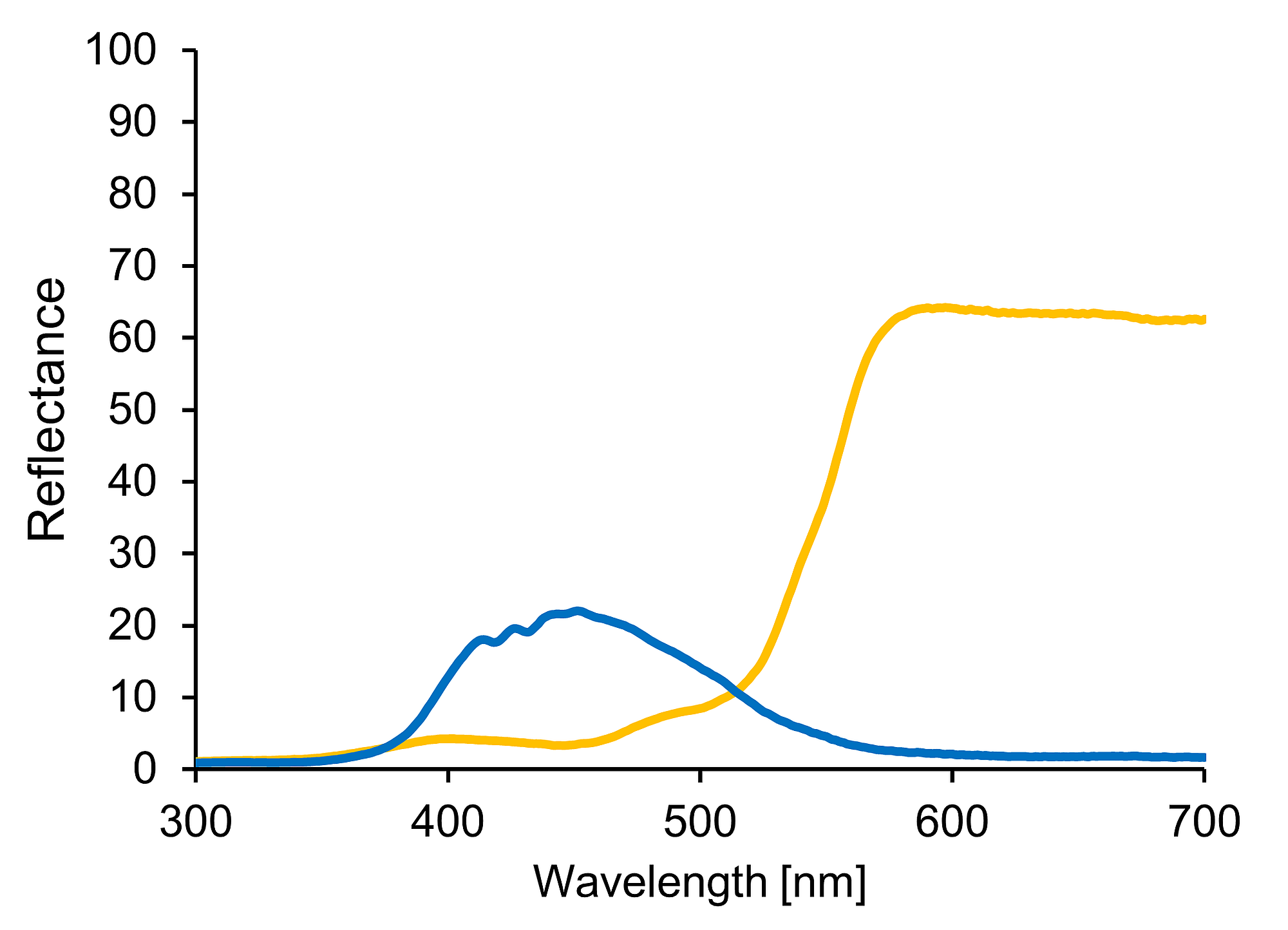

Supplement: FIGURE S1 — Spectral reflectance curves of colored feeders. [file Image_1.tif]
