# Supplementary material for: A Comparative Study of Food Source Selection in Stingless Bees and Honeybees: Scent Marks, Location, or Color
Source: Front Plant Sci. 2020 May 6;11:516. doi: 10.3389/fpls.2020.00516 (PMC7218124; doi:10.3389/fpls.2020.00516)
Supplement: TABLE S1 — Position of feeders in training and test phase. The order of tests was pseudo-randomized to ensure no influence of test order on the decisions of workers. Two experimental trials were conducted comprising four choice experiments each. C1–C4 are the tests which focused on blue color, while the tests C5–C8 focused on yellow color. SM1–3 = are tests which analyzed the impact of scent marks; m = marked feeder; u = unmarked feeder; C1–8 = are the tests analyzing the impact of color; b = blue; y = yellow; site 1 = 15 m distance to the hive; site 2 = 17 m distance to the hive. [file Table_1.pdf]

Test	Training site	Test site 1	Test site 2
SM1	site 1 (m)	m	u
SM2	site 2 (m)	u	m
SM3	site 1 (m)	u	m
C1	site 1 (b)	b	y
C2	site 2 (b)	y	b
C3	site 1 (b)	y	b
C4	site 1 (y)	y	b
C5	site 1 (y)	y	b
C6	site 2 (y)	b	y
C7	site 1 (y)	b	y
C8	site 1 (b)	b	y
